# Leaf Functional Traits of Two Species Affected by Nitrogen Addition Rate and Period Not Nitrogen Compound Type in a Meadow Grassland

**DOI:** 10.3389/fpls.2022.841464

**Published:** 2022-02-28

**Authors:** Lu Sun, Guojiao Yang, Yang Zhang, Siqi Qin, Jibin Dong, Yangzhe Cui, Xiao Liu, Peiming Zheng, Renqing Wang

**Affiliations:** ^1^School of Life Sciences, Institute of Ecology and Biodiversity, Shandong University, Jinan, China; ^2^Shandong Provincial Engineering and Technology Research Center for Vegetation Ecology, Shandong University, Jinan, China; ^3^Qingdao Forest Ecology Research Station of National Forestry and Grassland Administration, Qingdao, China; ^4^College of Ecology and Environment, Hainan University, Haikou, China

**Keywords:** leaf functional traits, meadow steppe, nitrogen compound type, nitrogen deposition, nitrogen addition period

## Abstract

Plasticity of plant functional traits plays an important role in plant growth and survival under changing climate. However, knowledge about how leaf functional traits respond to the multi-level N addition rates, multiple N compound and duration of N application remains lacking. This study investigated the effects of 2-year and 7-year N addition on the leaf functional traits of *Leymus chinensis* and *Thermopsis lanceolata* in a meadow grassland. The results showed that the type of N compounds had no significant effect on leaf functional traits regardless of duration of N application. N addition significantly increased the leaf total N content (LN) and specific leaf area (SLA), and decreased the leaf total P content (LP) and leaf dry matter content (LDMC) of the two species. Compared with short-term N addition, long-term N addition increased LN, LP, SLA, and plant height, but decreased the LDMC. In addition, the traits of the two species were differentially responsive to N addition, LN and LP of *T. lanceolata* were consistently higher than those of *L. chinensis.* N addition would make *L. chinensis* and *T. lanceolata* tend to “quick investment-return” strategy. Our results provide more robust and comprehensive predictions of the effects of N deposition on leaf traits.

## Introduction

Nitrogen (N) deposition in the global atmosphere has increased dramatically as a result of increased human activities, particularly the burning of fossil fuels and the development of intensive agriculture (Galloway et al., 2008; Yu et al., 2019). Nitrogen is a major limiting nutrient for plant growth in terrestrial ecosystems (Elser et al., 2007), with plant community structure and composition usually affected by changes in N availability (Clair et al., 2010). At present, increases in atmospheric N deposition have attracted the attention of researchers, it has been shown that increased N deposition has led to various ecological problems (Suding et al., 2005; Tian et al., 2020). For example, long-term N application can drive declines in plant diversity (Stevens et al., 2004; Clark and Tilman, 2008; Midolo et al., 2019), and long-term N addition is more likely to lead to other nutrient limitations [e.g., phosphorus (P)] in terrestrial ecosystems (Su et al., 2021).

Plant functional traits are important for exploring the relationship between plants and the environment, which provide a basis for predicting the response of ecosystems in the context of global change (Wright et al., 2010; Li et al., 2015; Liu et al., 2021b). Previous studies have suggested that plant functional traits are key to elucidating the mechanisms that drive the responses of plant communities and ecosystems to resource addition (McGill et al., 2006; Suding and Goldstein, 2008). Plant functional traits are often associated with the environment (Liu et al., 2020, 2021c). It is recognized that N deposition can alter leaf chemical, morphological, and physiological traits (Yan et al., 2012; Zhang et al., 2018). Previous studies found that increased N deposition increases the N concentration in green and senesced plant leaf tissue and also changes the stoichiometric ratio of plant leaves (Xia and Wan, 2008; Cory et al., 2011). Nitrogen addition also changes the specific leaf area, plant height, and dry matter content of leaves (Zheng et al., 2017). These results all show that leaf functional traits are closely related to N deposition. The nutritional status of plants and the response of leaf stoichiometric ratios to N enrichment are essential for elucidating how plants adapt to human disturbances (He et al., 2008; Elser et al., 2010).

Atmospheric N deposition involves a complex of N compounds (Galloway, 1995), which is mainly composed of ammonium (NHx) and nitrate (NOy; Yu et al., 2019). Some recent observations have shown differences in the response of soils and plants to different N compound addition type. Across Chinese grasslands, Liu et al. found that declines in soil pH varied with the N compound type (Liu et al., 2021a). In addition, a meta-analysis found that growth of terrestrial plants was enhanced more by NH4^+^-N than NO3^–^-N addition (Yan et al., 2019). In addition, the divergent responses of plant to N addition have been ascribed the response of species and ecosystems will be differential to the duration of N addition (Bobbink et al., 2010). Species abundance responded contradictorily to short-term and long-term N addition (Zheng et al., 2019; Yang et al., 2021), and N-induced P limitation also changed with the duration of N addition (Chen et al., 2020). Therefore, the effects of plant functional traits may also depend on the duration of N enrichment. These findings suggest that different N compounds have different effects on soil properties, which may result in different changes in plants. However, the impacts of different N compounds and the duration of N addition on leaf functional traits within the same location is still lacking.

In the present study, three N compounds and six N addition rates were selected to test the effect of N deposition on the leaf functional traits of two typical meadow plants, *Leymus chinensis* and *Thermopsis lanceolata*, based on the length of the fertilization period. We were particularly interested to examine: (1) How do plant leaf functional traits respond to different N addition gradients among different types of N compound? (2) Under the same N addition gradient treatment, are there differences in the response of plant leaf functional traits to short- and long-term N deposition?

## Materials and Methods

### Study Site

The study was conducted at the Erguna Forest-Steppe Ecotone Ecosystem Research Station (N50°10′46.1″, E119°22′56.4″). It is located in HulunBuir City, Inner Mongolia Autonomous Region. The soil is classified as chernozem according to the FAO classification. The region has a cold temperate continental climate with an average annual temperature of −1.59°C and an annual rainfall of 336.5 mm (2000–2020). The rainfall of the sample site was 148.2 mm in 2015 and 139.8 mm in 2020 according to the measurement of the research station. The background value of N deposition in this area is relatively low (Jia et al., 2014), which is conducive to simulation experiments of N deposition. Two dominant species, the C_3_ perennial rhizome grass *L. chinensis* and C_3_ perennial legume *T. lanceolata*, which together account for more than 50% of the peak community aboveground biomass, were used as our model plant species.

### Experimental Design

The grassland of the experiment site has been fenced since 2013 to prevent large animals, such as cattle and sheep, from eating and trampling the vegetation. The N addition experiment was established in 2014. All experimental plots (10 m × 10 m) had similar topography and land-use history. A completely randomized block design was used, and a 1-m buffer area was established between the plots. Three N compounds were selected: ammonium nitrate (NH_4_NO_3_), ammonium sulfate [(NH_4_)_2_SO_4_], and ammonium hydrogen carbonate (NH_4_HCO_3_). Each N fertilizer was applied at six concentrations: 0, 2, 5, 10, 20, and 50 g N m^–2^ yr^–1^. There were 18 treatments, with each treatment replicated eight times, resulting in a total of 144 plots. The N compounds were applied annually in late May. In order to ensure that the N compounds were spread evenly in each plot, they were combined with baked fine sand.

### Sampling and Leaf Functional Trait Determination

Plant sampling was conducted during the season of vigorous plant growth (August) in 2015 (short term) and 2020 (long term). These sampling dates corresponded to two years in the short-term experiment and seven years in the long-term experiment. Plant height (H) was obtained based on the mean of five healthy individuals at every site. Ten to 15 complete, healthy plants of each species were harvested, placed in sealed bags, and temporarily stored in an insulation bucket in iceboxes. After sampling was completed, the sample was immediately brought to the laboratory to reduce the loss of water dispersion from the plant and to avoid leaf shrinkage.

In the laboratory, all samples were soaked in water for 6 h to ensure full rehydration. Each leaf was then cut from the stem and gently dried with tissue paper before measurement. Water-saturated leaf and stem mass were weighed. The leaves used to measure the leaf area are scanned with a scanner after being weighed (Canon LiDE120), and then the leaf area was determined by the ImageJ software^1^. All leaves were then dried for 48 h at 65°C and then weighed. The leaf dry matter content (LDMC), specific leaf area (SLA), and stem-leaf ratio (S:L) were calculated. The leaf total N content (LN) was determined by the Kjeldahl apparatus (K9860, Hanon, Dezhou, China) after extraction with sulfuric acid, and the leaf total P content (LP) was determined by a UV-spectrophotometer (UA-5500, METASH, Shanghai, China) with the wavelength set to 700 nm. The N to P ratio (N:P) was calculated as the ratio of the total N content of the leaf to the total P content. All trait measurements were based on previous standardized measurements (Cornelissen et al., 2003; Pérezharguindeguy et al., 2013).

### Statistics

The N compound type was used as a categorical variable with three levels: NH_4_HCO_3_ (AC), NH_4_NO_3_ (AN), and (NH_4_)_2_SO_4_ (AS). The different sampling years were also categorical variables with two levels: 2015 (short term) and 2020 (long term). We used four-way ANOVA to test the effects of N addition rates, N compounds type, period, species, and their interactions on leaf chemical and morphological traits. The effects of different N addition rates on leaf chemical and morphological traits were tested using one-way ANOVA, followed by *post hoc* tests [Tukey’s honest significance difference (HSD)] in periods of treatment for each N compound type, and the effect of year was tested by *t*-tests in each N addition rate of each N compound type. When necessary, data were natural log-transformed to meet the normality assumption of ANOVA and *t*-tests (include SLA). The variation in plant leaf functional traits was analyzed based on differences by terms (N addition time), N compound type and N addition rates using PERMANOVA (999 permutations, Adonis function) and visualized by principal component analysis (PCA). In order to determine whether the relationship between the leaf functional traits of the two species in different years had changed, we used the ggpairs function in the Ggally package for plotting and calculated the correlation and significance between each two based on Pearson’s correlation analysis (Emerson et al., 2013). All statistical analyses were performed using R software 4.0.3 (R Core Team 2019^2^).

## Results

### Effects of N Addition Rate, N Compound Type, and Period on the Chemical Traits in the Plant Leaves

The results showed that the N addition rate, species and period had significant effects on the LN, LP and N:P of the two plants (*P* < 0.01), while N compound type had no significant effect (Table 1). The N addition rate and period had an interactive effect on the LN, LP and N:P of the two plants (*P* < 0.01, Table 1).

**TABLE 1:** Results (*F* values) of a repeated-measures ANOVA for the effects of N addition rate (R), N compounds type (T), period (Pr), species (S) and their interactions on leaf nitrogen content (LN), leaf phosphorus content (LP), leaf N content and leaf P content ratio (N:P), specific leaf area (SLA), leaf-stem ratio (S:L), leaf dry matter content (LDMC), and plant height (H) of *Leymus chinensis* and *Thermopsis lanceolata*.

	Df	LN	LP	N:P	Log_10_(SLA)	S:L	LDMC	H
R	5	31.10***	6.69***	10.53***	4.17**	3.26**	2.43*	10.89***
T	2	2.72^ns^	0.88^ns^	2.73^ns^	0.23^ns^	1.09^ns^	3.50*	0.62^ns^
Pr	1	1556.00***	1328.34***	243.87***	686.11***	79.68***	522.70***	2123.17***
S	1	1328.96***	244.22***	70.55***	878.22***	621.18***	1906.96***	4310.95***
R × T	10	0.75^ns^	0.84^ns^	0.52^ns^	1.02^ns^	0.77^ns^	0.56^ns^	0.57^ns^
R × Pr	5	9.20***	6.82***	0.55^ns^	6.37***	2.10^ns^	5.01***	9.65***
T × Pr	2	0.66^ns^	0.56^ns^	0.90^ns^	0.15^ns^	0.28^ns^	0.60^ns^	2.35^ns^
R × S	5	5.01***	1.82^ns^	3.08**	3.16**	1.13^ns^	1.91^ns^	2.85*
T × S	2	1.66^ns^	0.07^ns^	0.13^ns^	0.66^ns^	2.66^ns^	1.43^ns^	1.69^ns^
Pr × S	1	13.76***	148.70***	131.81***	205.23***	86.12***	35.91***	515.38***
R × T × Pr	10	0.68^ns^	1.24^ns^	1.11^ns^	1.21^ns^	0.71^ns^	1.65^ns^	0.59^ns^
R × T × S	10	0.41^ns^	0.88^ns^	0.43^ns^	0.54^ns^	1.02^ns^	1.52^ns^	0.79^ns^
R × Pr × S	5	1.31^ns^	2.81*	0.85^ns^	2.44*	2.17^ns^	0.56^ns^	2.26*
T × Pr × S	2	0.45^ns^	0.58^ns^	0.89^ns^	0.31^ns^	0.91^ns^	1.40^ns^	0.31^ns^
R × T × Pr × S	10	0.70^ns^	0.59^ns^	0.76^ns^	0.93^ns^	1.20^ns^	0.93^ns^	1.53^ns^

Asterisks denote significant levels: ns, P > 0.05; *, P ≤ 0.05; **, P ≤ 0.01; and ***, P ≤ 0.001, respectively.

**FIGURE 1:**
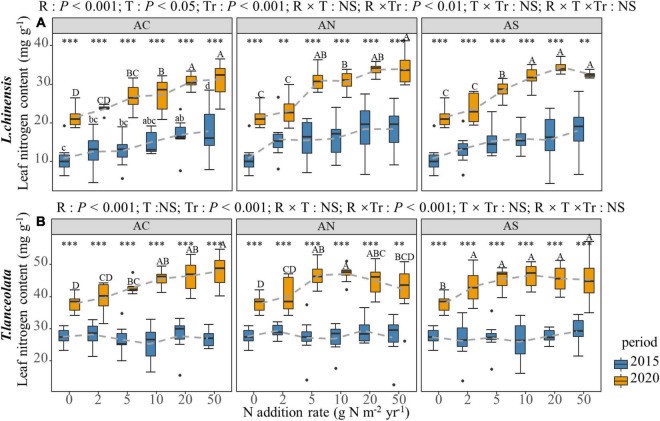
Effects of short- and long-term nitrogen deposition on LN of two species: **(A)** *Leymus chinensis*; **(B)** *Thermopsis lanceolata*. The three-way ANOVA was used to test the effects of Pr, T, and R on LN. The effects of the nitrogen compound type alone and with N addition rate and year on LN are not significant (NS, *P* > 0.05), thus one-way ANOVA (Tukey’s HSD) and *t*-tests are used to test the effects of N addition rate and year on LN. Different lower-case and upper-case letters indicate significant differences (*P* < 0.05) in LN among nitrogen addition rate in the 2015 and 2020 plots, respectively. The asterisk above the picture indicates the significant difference between the two years under each nitrogen addition rate. Note: ^**^P < 0.01; ^***^P < 0.001. CK: non-fertilizer control = 0, N1 = 2, N2 = 5, N3 = 10, N4 = 20, and N5 = 50 kg N ha^–1^ yr^–1^; AC (NH_4_HCO_3_), AN (NH_4_NO_3_), AS [(NH_4_)_2_SO_4_]. The gray dashed line is the connection of the mean value.

Nitrogen addition significantly increased the LN in both species. However, under high N addition (20 and 50 g N m^–2^ yr^–1^), the LN of the two species showed a slowing down or a slight downward trend with increasing N addition (Figure 1). The increasing trend of LN under long-term N addition was significantly higher than that under short-term N addition (Figure 1). Under both short- and long-term N addition treatments, the LN of the two species showed significant differences at each N addition rate of the two periods. Similar results were detected for all three N addition types.

**FIGURE 2:**
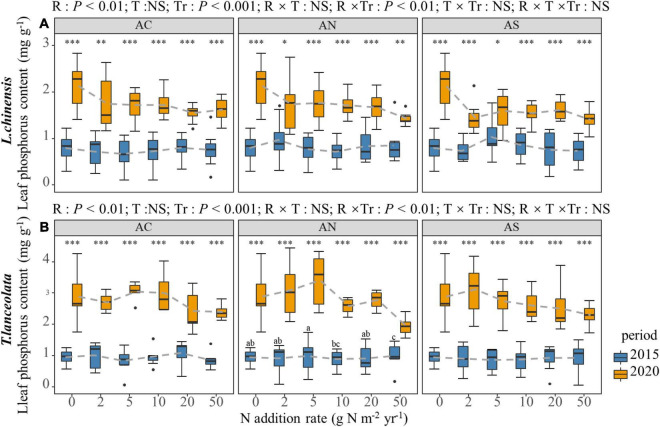
Effects of short- and long-term nitrogen deposition on LP of two species: **(A)** *Leymus chinensis*; **(B)** *Thermopsis lanceolata*. The three-way ANOVA was used to test the effects of Pr, T and R on LP. The effects of the nitrogen compound type alone and with nitrogen addition rate and year on LP are not significant (NS, *P* > 0.05), thus one-way ANOVA (Tukey’s HSD) and *t*-tests are used to test the effects of nitrogen addition rate and year on LP. Different lower-case and upper-case letters indicate significant differences (*P* < 0.05) in LN among nitrogen addition rate in the 2015 and 2020 plots, respectively. The asterisk above the picture indicates the significant difference between the two years under each nitrogen addition rate. Note: *P < 0.05; ^**^P < 0.01; ^***^P < 0.001. CK: non-fertilizer control = 0, N1 = 2, N2 = 5, N3 = 10, N4 = 20, and N5 = 50 kg N ha^–1^ yr^–1^; AC (NH_4_HCO_3_), AN (NH_4_NO_3_), AS [(NH_4_)_2_SO_4_]. The gray dashed line is the connection of the mean value.

The LP of the two species did not show a significant trend in N addition rate with the addition of various N compounds (Figure 2). The LP of *L. chinensis* under N addition was only marginally lower than the control. The LP of the two species showed significant differences at each N addition rate of the two periods. The N content under long-term N addition was significantly higher than under short-term N addition (Figure 2, *P* < 0.05).

**FIGURE 3:**
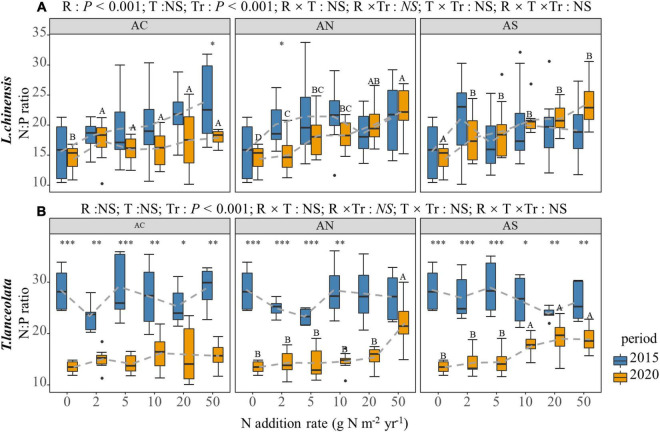
Effects of short- and long-term nitrogen deposition on N:P of two species: **(A)** *Leymus chinensis*; **(B)** *Thermopsis lanceolata*. The three-way ANOVA was used to test the effects of Pr, T and R on N:P. The effects of the nitrogen compound type alone and with nitrogen addition rate and year on N:P are not significant (NS, *P* > 0.05), thus one-way ANOVA (Tukey’s HSD) and *t*-tests are used to test the effects of nitrogen addition rate and year on N:P. Different lower-case and upper-case letters indicate significant differences (*P* < 0.05) in LN among nitrogen addition rate in the 2015 and 2020 plots, respectively. The asterisk above the picture indicates the significant difference between the two years under each nitrogen addition rate. Note: *P < 0.05; ^**^P < 0.01; ^***^P < 0.001. CK: non-fertilizer control = 0, N1 = 2, N2 = 5, N3 = 10, N4 = 20, and N5 = 50 kg N ha^–1^ yr^–1^; AC (NH_4_HCO_3_), AN (NH_4_NO_3_), AS [(NH_4_)_2_SO_4_]. The gray dashed line is the connection of the mean value.

The N:P of the two species differed significantly in response to the N addition rate and period (Figure 3). The N:P of the leaf of *L. chinensis* significantly increased under long-term N addition (Figure 3A, *P* < 0.05), while that was not significant under short-term N addition. Furthermore, the difference between the two periods was not significant (Figure 3A, *P* > 0.05). The change trend of the N:P in the *T. lanceolata* leaves was not obvious with N addition. The N:P under long-term N addition was significantly lower than under short-term N addition (Figure 3B).

### Effects of N Addition Rate, N Compound Type, and Period on the Morphological Traits in Plant Leaves

According to four-way ANOVA, the N addition rate, species and period had significant effects on the LDMC, S:L, H and SLA of the two plants (*P* < 0.05), while N compound type had no significant effect, except for the LDMC (Table 1). The N addition rate and period had an interactive effect on the LDMC, S:L, H and SLA of the two plants (*P* < 0.01, Table 1).

**FIGURE 4:**
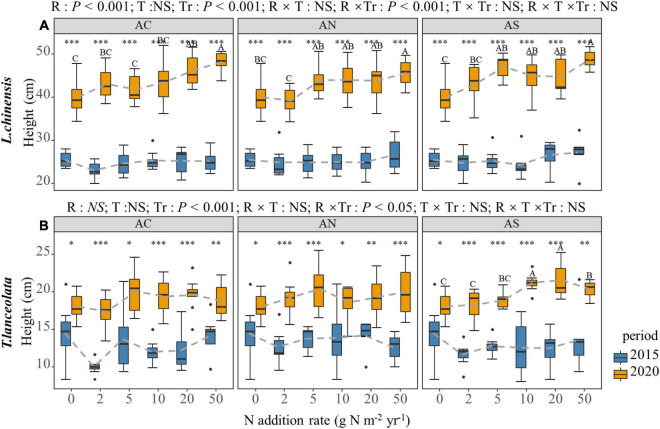
Effects of short- and long-term nitrogen deposition on H of two species: **(A)** *Leymus chinensis*; **(B)** *Thermopsis lanceolata*. The three-way ANOVA was used to test the effects of Pr, T and R on H. The effects of the nitrogen compound type alone and with nitrogen addition rate and year on H are not significant (NS, *P* > 0.05), thus one-way ANOVA (Tukey’s HSD) and *t*-tests are used to test the effects of nitrogen addition rate and year on H. Different lower-case and upper-case letters indicate significant differences (*P* < 0.05) in LN among nitrogen addition rate in the 2015 and 2020 plots, respectively. The asterisk above the picture indicates the significant difference between the two years under each nitrogen addition rate. Note: *P < 0.05; ^**^P < 0.01; ^***^P < 0.001. CK: non-fertilizer control = 0, N1 = 2, N2 = 5, N3 = 10, N4 = 20, and N5 = 50 kg N ha^–1^ yr^–1^; AC (NH_4_HCO_3_), AN (NH_4_NO_3_), AS [(NH_4_)_2_SO_4_]. The gray dashed line is the connection of the mean value.

With the increase in N addition, the H of *L. chinensis* showed a significant upward trend under long-term N addition (*P* < 0.05), but there was no significant change under short-term N addition (Figure 4A). With the exception of the AS treatment under long-term N addition, the response of *T. lanceolata* H to N addition rate was not significant (Figure 4B, *P* < 0.05). There was a significant inter-annual difference in the H of the two species. The plant height under long-term N addition was significantly higher than under short-term N addition in both species (Figure 4, *P* < 0.05).

**FIGURE 5:**
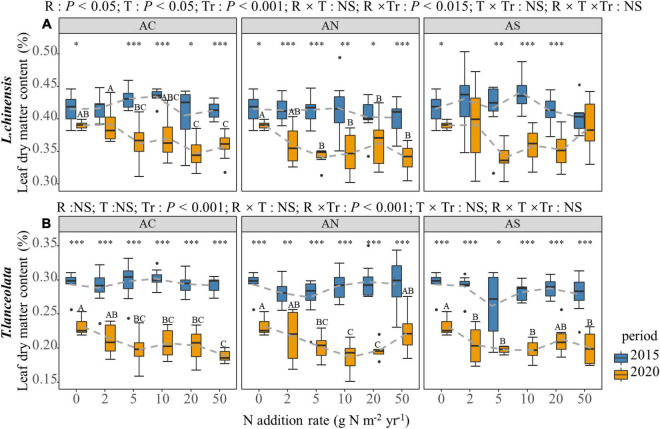
Effects of short- and long-term nitrogen deposition on LDMC of two species: **(A)** *Leymus chinensis*; **(B)** *Thermopsis lanceolata*. The three-way ANOVA was used to test the effects of Pr, T and R on LDMC. The effects of the nitrogen compound type alone and with nitrogen addition rate and year on LDMC are not significant (NS, *P* > 0.05), thus one-way ANOVA (Tukey’s HSD) and *t*-tests are used to test the effects of nitrogen addition rate and year on LDMC. Different lower-case and upper-case letters indicate significant differences (*P* < 0.05) in LN among nitrogen addition rate in the 2015 and 2020 plots, respectively. The asterisk above the picture indicates the significant difference between the two years under each nitrogen addition rate. Note: *P < 0.05; ^**^P < 0.01; ^***^P < 0.001. CK: non-fertilizer control = 0, N1 = 2, N2 = 5, N3 = 10, N4 = 20, and N5 = 50 kg N ha^–1^ yr^–1^; AC (NH_4_HCO_3_), AN (NH_4_NO_3_), AS [(NH_4_)_2_SO_4_]. The gray dashed line is the connection of the mean value.

The N addition rate significantly affected the LDMC of the two species under long-term N addition (Figure 5, *P* < 0.05), but there was no significant difference in short-term N addition. The LDMC showed a gradual decrease with increased N addition. However, under high N treatment (20, 50 g Nm^–2^yr^–1^), LDMC demonstrated a slowing-down or slight upward trend (Figure 5). In period of the response between years, the LDMC of plants under long-term N addition was significantly lower than under short-term N addition (Figure 5, *P* < 0.05).

**FIGURE 6:**
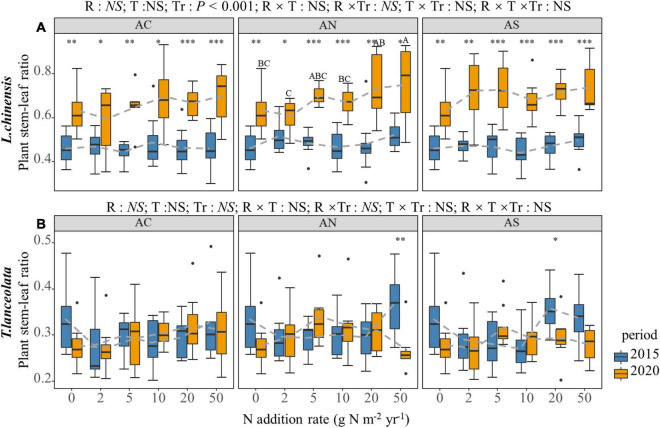
Effects of short- and long-term nitrogen deposition on S:L of two species: **(A)** *Leymus chinensis*; **(B)** *Thermopsis lanceolata*. The three-way ANOVA was used to test the effects of Pr, T and R on S:L. The effects of the nitrogen compound type alone and with nitrogen addition rate and year on S:L are not significant (NS, *P* > 0.05), thus one-way ANOVA (Tukey’s HSD) and *t*-tests are used to test the effects of nitrogen addition rate and year on S:L. Different lower-case and upper-case letters indicate significant differences (*P* < 0.05) in LN among nitrogen addition rate in the 2015 and 2020 plots, respectively. The asterisk above the picture indicates the significant difference between the two years under each nitrogen addition rate. Note: * *P* < 0.05; ^**^ *P* < 0.01; ^***^ *P* < 0.001. CK: non-fertilizer control = 0, N1 = 2, N2 = 5, N3 = 10, N4 = 20, and N5 = 50 kg N ha^–1^ yr^–1^; AC (NH_4_HCO_3_), AN (NH_4_NO_3_), AS [(NH_4_)_2_SO_4_]. The gray dashed line is the connection of the mean value.

The S:L of the two species demonstrated a significantly different response to the N addition rate and period (Figure 6). The S:L of *L. chinensis* showed an upward trend under long-term N addition (Figure 6A). The *t*-test showed that the response of *L. chinensis* S:L to period was significant (Figure 6A, *P* < 0.05). The S:L under long-term N addition was significantly higher than that under short-term N addition, while the response of *T. lanceolata* S:L to period was not significant (Figure 6B).

**FIGURE 7:**
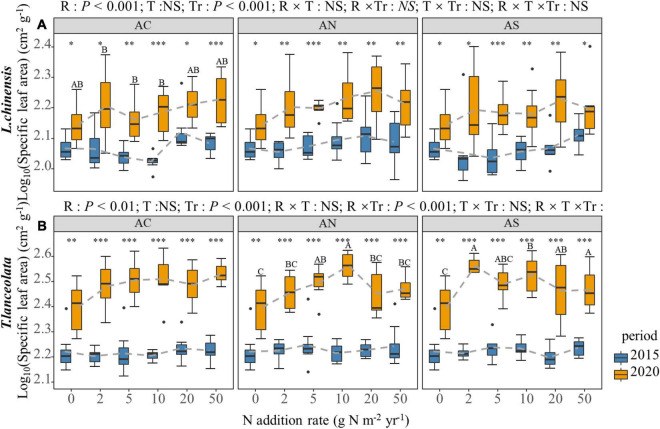
Effects of short- and long-term N deposition on SLA of two species: **(A)** *Leymus chinensis*; **(B)** *Thermopsis lanceolata*. The three-way ANOVA was used to test the effects of Pr, T and R on SLA. In order to make the data more stable, the value of the SLA is the logarithm of the original data. The effects of the nitrogen compound type alone and with nitrogen addition rate and year on SLA are not significant (NS, *P* > 0.05), thus one-way ANOVA (Tukey’s HSD) and *t*-tests are used to test the effects of nitrogen addition rate and year on SLA. Different lower-case and upper-case letters indicate significant differences (*P* < 0.05) in LN among nitrogen addition rate in the 2015 and 2020 plots, respectively. The asterisk above the picture indicates the significant difference between the two years under each nitrogen addition rate. Note: * *P* < 0.05; ^**^ *P* < 0.01; ^***^ *P* < 0.001. CK: non-fertilizer control = 0, N1 = 2, N2 = 5, N3 = 10, N4 = 20, and N5 = 50 kg N ha^–1^ yr^–1^; AC (NH_4_HCO_3_), AN (NH_4_NO_3_), AS [(NH_4_)_2_SO_4_]. The gray dashed line is the connection of the mean value.

Nitrogen addition increased the leaf SLA of the two species, especially under long-term N addition (Figure 7). However, under high N addition (20 and 50 g Nm^–2^yr^–1^), the leaf SLA of the two species slowed down or decreased slightly with the increase in N addition (Figure 7). The leaf SLA under long-term N addition was significantly higher than under short-term N addition (Figure 7, *P* < 0.05).

### Effects of Long- and Short-Term N Deposition on the Correlation of Plant Traits

**FIGURE 8:**
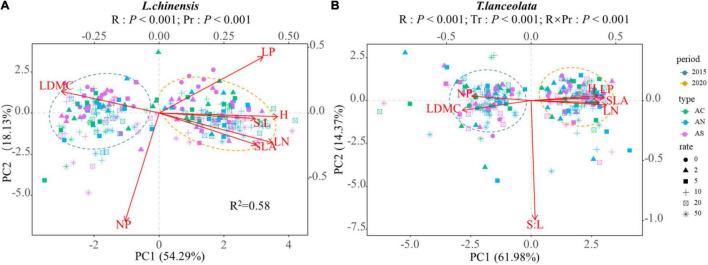
Principal components analysis (PCA) for all leaf functional traits of two species across different N addition rates and different periods: **(A)** *Leymus chinensis*; **(B)** *Thermopsis lanceolata*. The length of a variable vector in the representation space is indicative of the variable’s level of contribution. Different shapes represent different nitrogen addition rates, and different colors represent different N compound type. The two ovals are the distribution ranges of plant traits in two periods respectively.

The PCA of all variables showed that short- and long-term N addition resulted in obvious differences in the spatial aggregation of leaf functional traits, and the response of leaf functional traits to N addition rate and period differed (Figure 8). Compared with short-term N addition, *L. chinensis* had higher H, LN, LP, S:L, and SLA and lower LDMC under long-term N addition (Figure 8A), and *T. lanceolata* had higher H, LN, LP, and SLA and lower LDMC and N:P. There was a significant correlation between the leaf functional traits of *L. chinensis* and *T. lanceolata* under short- and long-term N addition (Supplementary Figures 1–4, *P* < 0.05). Compared with short-term N addition, long-term N addition had more pairs of traits that were significantly related (Supplementary Figures 1–4). Under long-term N addition, the H and LP, H and N:P, and LP and LN of both *L. chinensis* and *T. lanceolata* showed a significant change trend that was opposite to that under short-term N addition (Supplementary Figures 1–4, *P* < 0.05), which indicated that long-term N addition will alter the relationships of some plant functional traits.

## Discussion

### Effects of N Addition Rate, N Compound Type, and Period on the Chemical Traits in the Plant Leaves

Leaf N and P concentrations and the N:P stoichiometric ratios of the two species in the meadow steppe showed significant changes in response to the N addition rate and period. N addition led to an increase in the LN of the two species (Figure 1), which corroborates the results of previous studies (Huang et al., 2012; Lü et al., 2013). Nitrogen addition caused an increase in soil inorganic N concentrations, which subsequently enhanced the N concentrations in the leaves (Shi et al., 2020). Moreover, N addition increased the re-absorption rate of plant N (Lü et al., 2012; Cao et al., 2021), which in turn increased the plant N content. However, the LN did not always increase with the increase in the N addition rate. At high N addition rate (20 and 50 g Nm^–2^yr^–1^), the LN decreased slightly, which may be caused by the ammonium toxicity caused by excessive N fertilizer (Van den Berg et al., 2005; Phoenix et al., 2012). Ammonium toxicity effects mainly occur in plants that absorb ammonium ions more easily and exchange these ions with hydrogen ions and potassium plasma, thereby interfering with the cation balance in plants (Van den Berg et al., 2005).

Previous studies on the response of leaf P concentration to N addition obtained different conclusions, including negative (Menge and Field, 2007), neutral (Chen et al., 2015), and positive effects (Lü et al., 2013; Huang et al., 2018). In this work, N addition reduced the LP of *L. chinensis* leaves (Figure 2), resulting in a nutrient imbalance. N addition increases the biomass of the plant, and the plant will also grow higher under N addition, which leads to the dilution of the P concentration (Zhang et al., 2017). In order to maintain a stoichiometric balance, the demand for P increases as the N concentration of the leaves increases, and the release of available P from plants through soil formation is usually very slow (Houlton et al., 2008; Zhang et al., 2017). The *T. lanceolata* did not show a similar phenomenon, which may be due to the specific characteristics of the species (Mayor et al., 2014; Wan et al., 2019). The N and P concentrations were less influenced by N addition under short-term N addition than under long-term N addition, probably because of the timing of N application. Because as the time of nitrogen addition increases, the accumulated nitrogen in the soil increases (Sun et al., 2020).

The N-to-P ratio is a measure of plant growth limitation. Güsewell (2004) proposed an N:P > 20 and <10 as the evaluation index of plant N-P limitation. When N-to-P ratio > 20, this indicates that plant growth is limited by P, while N-to-P ratio < 10 indicates that plant growth is limited by N. N-to-P ratio of 10 – 20 indicates that plant growth was not limited or co-limited by N and P (Yan et al., 2017). In this study, the N:P of the *L. chinensis* leaves gradually increased with N addition rate in both periods, which indicated that growth gradually changed to P limitation (Figure 3A). The N:P of *T. lanceolata* under long-term N addition was significantly lower than under short-term N addition. According to the criteria proposed by Güsewell, the growth of *T. lanceolata* was mainly limited by P under short-term N addition, while it was limited by both N and P under long-term N addition. The results indicated that N addition could gradually change the elements involved in ecosystem limitations.

### Effects of N Addition Rate, N Compound Type, and Period on the Morphological Traits of the Plant Leaves

Nitrogen addition had a significant effect on SLA and LDMC in the plant leaves, which aligns with previous studies (Knops and Reinhart, 2000) and demonstrates that N addition influences these leaf functional traits. In this study, N addition increased the H of the plants, indicating that N addition promoted plant growth, the plant growth strongly affected by nitrogen limitation. The results showed that, N addition increased the SLA of the plant leaves and decreased the LDMC. From an inter-year perspective, long-term N application had higher SLA and lower LDMC compared with short-term N application. SLA and LDMC are key indicators of nutrient utilization strategies (Garnier et al., 2004). A high SLA and low LDMC represent rapid nutrient acquisition, which is conducive to the growth of plants in a nutrient-rich environment, while low SLA and high LDMC are suitable for plants in nutrient-poor environments. In the environment, plants adopt a conservative growth strategy (Wilson et al., 2010; Rose et al., 2013). Our results showed that N addition had a beneficial effect on the growth of *L. chinensis* and *T. lanceolata*. Taken together, our results suggest that the plasticity of plant leaf functional traits expressed allowed the plants to adapt to environment changes under increased N deposition (Agrawal, 2001).

Under the background of N addition, changes in plant leaf morphological traits represent a type of phenotypic plasticity. Although our results reflected that the trait plasticity of both plants changed in a favorable direction for plant growth, longer N deposition experiments are encouraged to understand whether N saturation of leaf traits would occur in future.

Our results suggest that the effects of different N compounds on leaf functional traits were not significant. As the N compounds selected in the experiment all have ammonium ions, this shows that different anions (SO_4_^–^, HCO_3_^–^, NO_3_^–^) have no significant effect on leaf functional traits.

### Effects of Long- and Short-Term N Deposition on the Correlation of Plant Traits

We found that under long-term and short-term N application, the effect of N addition rates on plant traits was similar. Wright et al. (2004) proposed the “leaf economic spectrum” (LES) and found that the traits of plant leaves are arranged in an orderly manner along a continuously changing combination spectrum of functional traits. The survival strategies of plants are divided into a “”quick investment-return” strategy and “slow investment-return” strategy. In our study, compared with short-term N addition, long-term N addition made plant leaves having higher LN, higher LP, higher plant height, lower LDMC, and higher SLA, indicating that N addition would result in *L. chinensis* and *T. lanceolata* having a tendency toward larger and thinner leaves and a “quick investment-return” strategy. Therefore, meadow grassland plants adapt to the long-term nitrogen deposition environment mainly through the distribution and regulation of resources. In addition, a fast strategy may be beneficial in N deposition condition because it allows plants to take advantage of high resource, thus increasing their probability of survival. We also found that the correlation between plant leaf functional traits changed under long-term N addition conditions. This is similar to previous studies by Wright and Cannon (2001) and Pensa et al. (2010). Our results suggest that long-term nitrogen application tends to lead to closer relationships between traits. In addition, the correlation of traits involved in the “leaf economic spectrum” tended to prevail under N addition, but the magnitude of the correlation coefficients did not remain constant, and even opposite relationships were observed. This suggests that the relationships between plant traits are not absolutely stable, but vary with the environment. Therefore, when we try to use the general correlation of leaf traits to predict other relatively difficult-to-measure traits from some of the easily measured traits, we need to take into account the environmental changes such as local climate and geographical characteristics.

## Conclusion

Our results revealed that N deposition will affect leaf functional traits of *L. chinensis* and *T. Lanceolata* in meadow grassland. The N compound type had little effect on the properties, while the N addition rates and the period significantly increased the LN, LP, and SLA, and decreased the LDMC of the plant leaves. The correlation between leaf traits would change with the duration of N addition. The effects of long-term N addition to plant leaf functional traits were significantly higher than short-term treatments, which indicated the cumulative effect of the N deposition. The response of *L. chinensis* and *T. Lanceolata* to the N addition tends to be a “quick investment-return” strategy. These results clearly showed that N deposition can promote plant height, which will increase their light competitiveness and provide advantages for their own growth. And it is necessary for us to use changes in the external environment as one of the factors of reference in the future when predicting traits using the universal relationships related to leaf traits.

## Data Availability Statement

The original contributions presented in the study are included in the article/Supplementary Material, further inquiries can be directed to the corresponding author.

## Author Contributions

PZ designed the research and secured funding. LS, GY, YZ, SQ, JD, YC, and XL contributed to the field and laboratory measurements. LS and GY analyzed the data. RW provided ideas for writing. LS wrote the manuscript that was intensively edited by all of the authors. All authors contributed to the article and approved the submitted version.

## Conflict of Interest

The authors declare that the research was conducted in the absence of any commercial or financial relationships that could be construed as a potential conflict of interest.

## Publisher’s Note

All claims expressed in this article are solely those of the authors and do not necessarily represent those of their affiliated organizations, or those of the publisher, the editors and the reviewers. Any product that may be evaluated in this article, or claim that may be made by its manufacturer, is not guaranteed or endorsed by the publisher.

## Footnotes

1
http://imagej.nih.gov/ij/

2
http://www.R-project.org
