# Supplementary material for: Leaf Functional Traits of Two Species Affected by Nitrogen Addition Rate and Period Not Nitrogen Compound Type in a Meadow Grassland
Source: Front Plant Sci. 2022 Feb 28;13:841464. doi: 10.3389/fpls.2022.841464 (PMC8918929; doi:10.3389/fpls.2022.841464)

Supplementary Material

# Supplementary Figures

**Supplementary Figure 1.** Pearson correlation analysis among the leaf functional traits of *Leymus chinensis* in 2015

This is a generalized pairs plot showing, below the diagonal, all the pairwise scatter plots comparing each pair of leaf functional traits. Above the diagonal are correlation statistics (Pearson's product-moment correlation r) and the significance of the least-squares regression (P value). Different colors represent different amounts of nitrogen addition. (Similarly hereinafter)


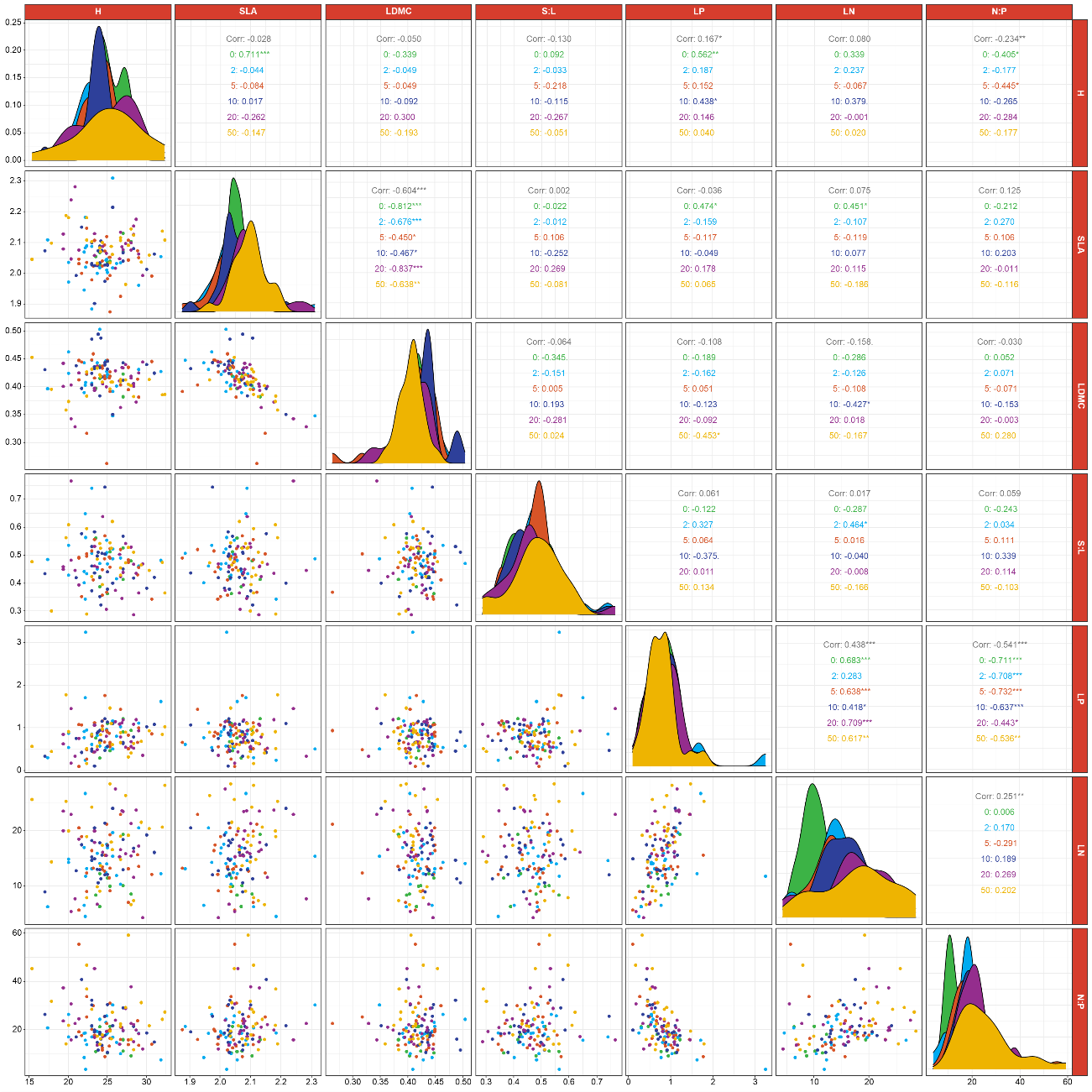


**Supplementary Figure 2.** Pearson correlation analysis among the leaf functional traits of *Leymus chinensis* in 2020


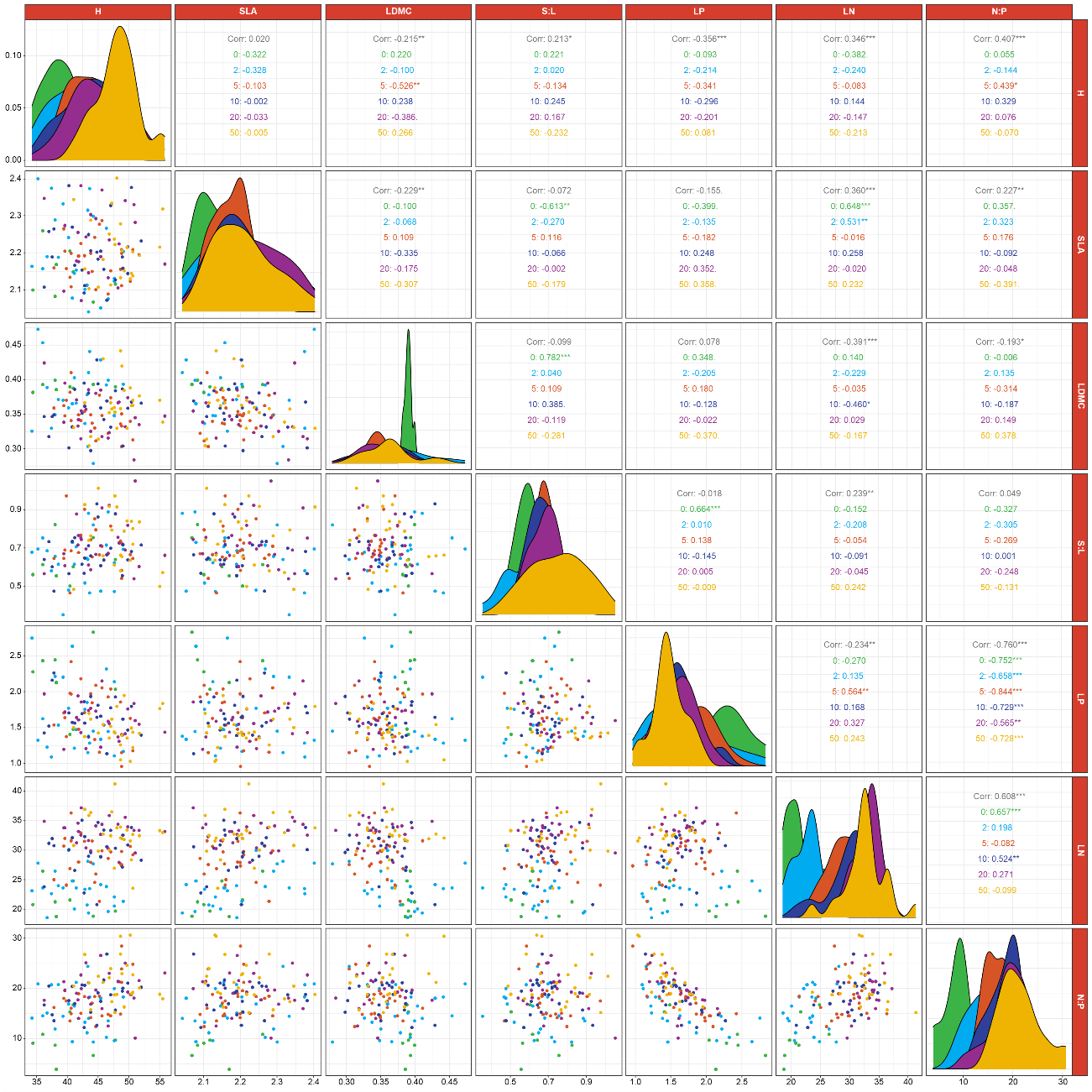


**Supplementary Figure 3.** Pearson correlation analysis among the leaf functional traits of *Thermopsis lanceolata* in 2015


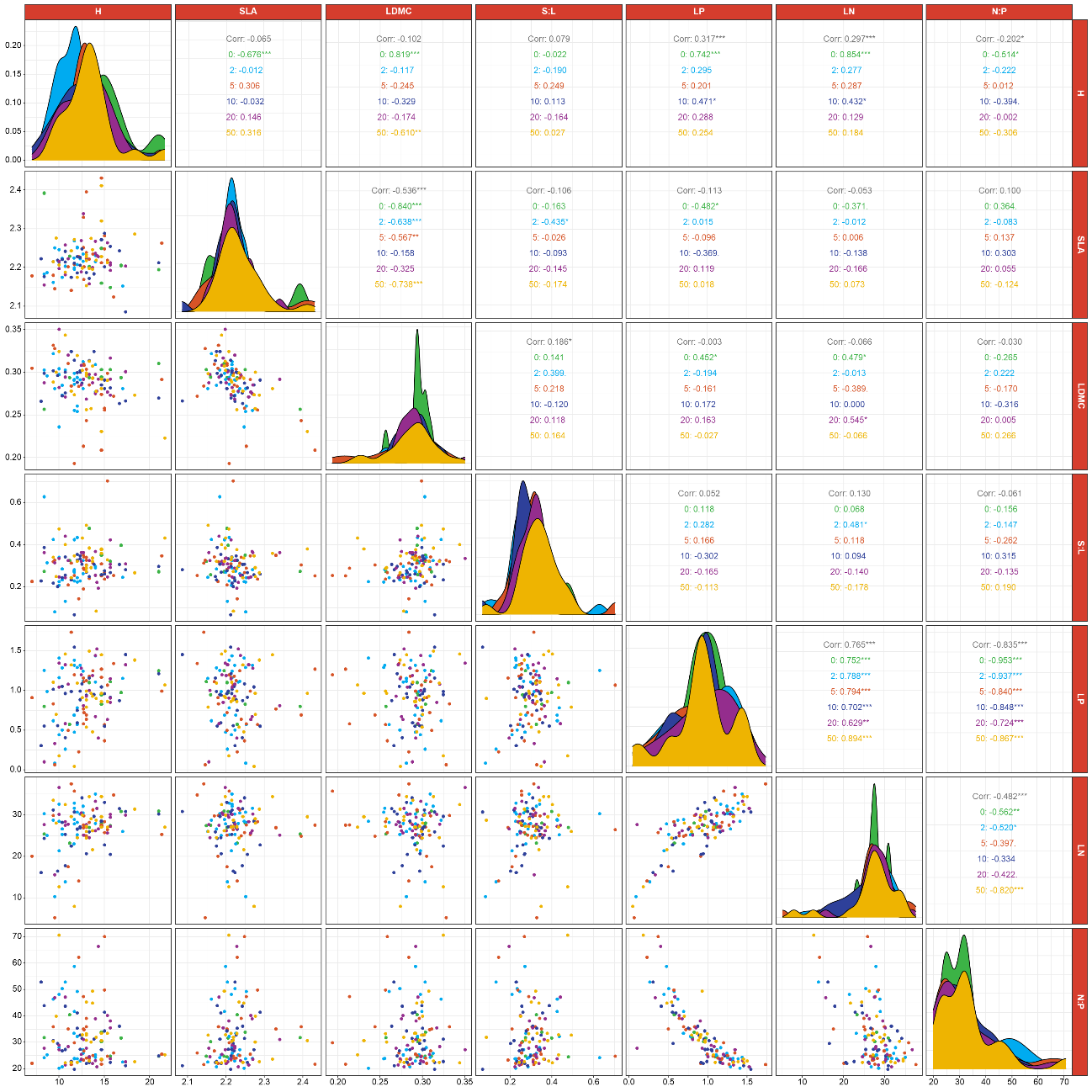


**Supplementary Figure 4.** Pearson correlation analysis among the leaf functional traits of *Thermopsis lanceolata* in 2020


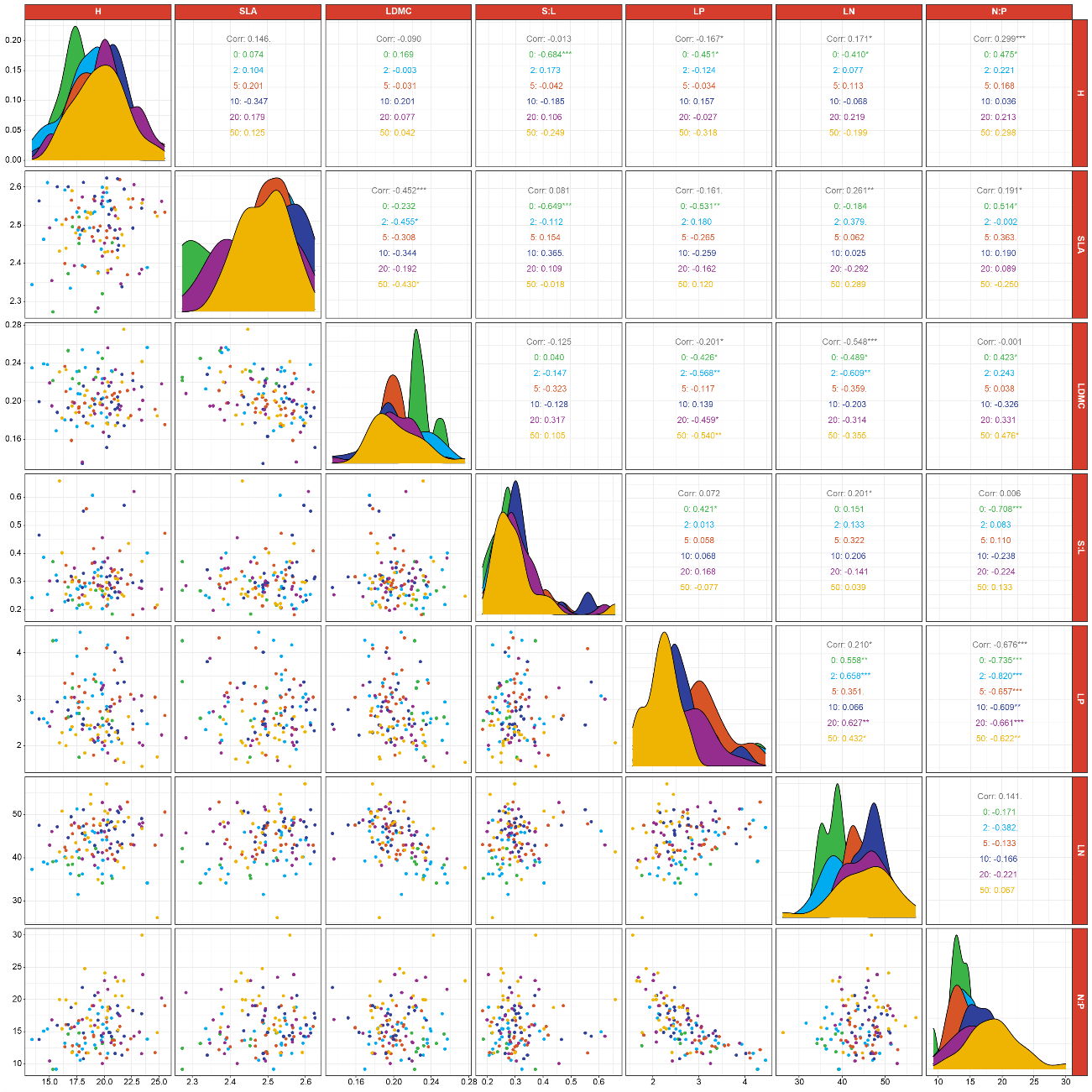

Supplement: Supplementary file 2 [file Data_Sheet_2.docx]
